# A laser emitting contact lens for eye tracking

**DOI:** 10.1038/s41598-020-71233-1

**Published:** 2020-09-09

**Authors:** A. Khaldi, E. Daniel, L. Massin, C. Kärnfelt, F. Ferranti, C. Lahuec, F. Seguin, V. Nourrit, J.-L. de Bougrenet de la Tocnaye

**Affiliations:** 1 Optics Department, IMT Atlantique, CS 83818, 29238 Brest CEDEX 3, France; 2 Electronic Department, IMT Atlantique, CS 83818, 29238 Brest CEDEX 3, France; 3 Microwave Department, IMT Atlantique, CS 83818, 29238 Brest CEDEX 3, France; 4 Lab-Sticc, UMR6285, IMT Atlantique, CS 83818, 29238 Brest CEDEX 3, France

**Keywords:** Engineering, Nanoscience and technology, Optics and photonics

## Abstract

In this paper, we present the first realisation and experimentation of a new eye tracking system using an infrared (iR) laser pointer embedded into a wireless smart contact lens. We denote this contact lens prototype as the cyclops lens, in reference to the famous hero of the X-Men comics. The full eye tracker device combines the smart contact lens and its eyewear, which provides a primary source of energy and the beam detection system. We detail the assembling and encapsulation process of the main functionalities into the contact lens and present how a gaze tracking system is achieved, compared to existing conventional eye-tracking ones. Finally, we discuss future technical improvements.

## Introduction

Knowing and analysing the gaze direction has become a key operation when using, for instance, augmented or virtual reality display systems for which it is useful to assess the attentional or cognitive load or to validate an operation by designating it by gaze^1–3^. Although a wide range of eye tracking techniques exist, these techniques are not always best suited to be used with an AR/VR headset. For instance, electro-oculography^4^ has limited accuracy, scleral-coils^5^ are uncomfortable requiring the eye to be anesthetized. Standard video-based techniques^3^ require a clear view of the eyes (e.g., glasses are an issue), small yet high aperture cameras and relatively high computing power, to provide a sufficiently high accuracy. As a result, the issue of integrating a performing eye tracker into an AR/VR headset is still an active research topic^6,7^.

In parallel, encapsulation of functions in a wireless contact lens has been made possible thanks to recent advances in technologies for manufacturing micro-scale optoelectronic components, as well as methods for assembling them onto separately-formed substrates^8,9^. As a result, a variety of electronic contact lenses have been proposed in the last two decades. Due to the nature of the lens, the applications concern mainly wearable smart sensors for health diagnostics^10,11^, the correction of vision or refractive errors^12^, gaze tracking^13^ or displays^14–17^. Some wireless contact lenses have been tested on persons (e.g. studies carried out at Moorfields Eye Hospital in London^18^) and have been commercialized in medical products (e.g., Sensimed^19^). However, their overall functions were relatively specific (e.g., intraocular pressure gauge (IOP) or glucose monitoring) they operated as interrogators and did not implement complex cognitive tasks. In this context, including a laser pointer directly on the eye could provide a potentially simpler and more compact solution to measure eye gaze compared to available technologies based mostly on image processing^20,21^. It would also allow demonstrating the use of an electronic contact lens as component of a more complex system (eye tracking).

The operating principle of the eye tracking system is described below. The idea to use a marker into a contact lens is not new^22^ and neither the integration of a light source into a contact lens. The integration of a micro-LED has already been demonstrated for other applications [e.g.^23^]. We propose here to use a vertical cavity surface emitting laser (VCSEL) because of the good ratio between the laser power and the power supplied as well as the very low divergence (few tens of mrad without any additional optics) which allows for a high quality beam spot (necessary here to get a good detection accuracy). Our goal was here to scale all the elements to design and realize a complete eye tracking system, showing how the spot emitted by a laser pointer can be detected correctly by the sensor (independently from the eye rotation) with a good accuracy, for a given distance between the eye and the eyewear, and in compliance with the security standards (optical and RF).

## Eyetracking operating principle

The idea here consists in encapsulating a laser pointer or a light emitting source^24^ into a contact lens to materialize the direction of gaze, therefore strongly simplifying its calculation. Most commercial eye trackers rely on imaging the eyes. This involves illuminating the eyes with IR light sources, recording several MB of information/s with fast cameras and processing this information to extract, from the pupil and corneal reflection position, the gaze direction at high frequencies. As stated previously, a number of factors such as iris pigmentation, wearing spectacles, sunlight can reduce such eye tracker performance. Other approaches such as electrooculography or lenses with magnetic coils exist but their implementation makes them suitable only for research or clinical studies^25^. Indeed, these two techniques, as the cyclops lens, require some preparation (fitting the lens, the electrodes or the coils) and are more invasive than the video based eye trackers. When compared to the scleral coil approach, the main advantage of the cyclops lens is that there are no external parts. All the electronics is embedded within the lens. Electrooculography can be useful in some context (e.g. when the eyes are closed) but offer limited resolution^3^ (~ 2°) and are sensitive to various factors (facial muscle activity, electrical interferences, etc.) that limit its use.

In our system, detecting the laser beam direction will directly provide the gaze direction. This can be achieved in different ways. A solution consists in using a 2D planar detector^26^ such as a position sensitive detector (PSD). In this case, the remote control system is replaced by the eye, provided that a collimated laser with a sufficient light power to be detected by the PSD is used, in case this one is far from the lens.

**Figure 1:**
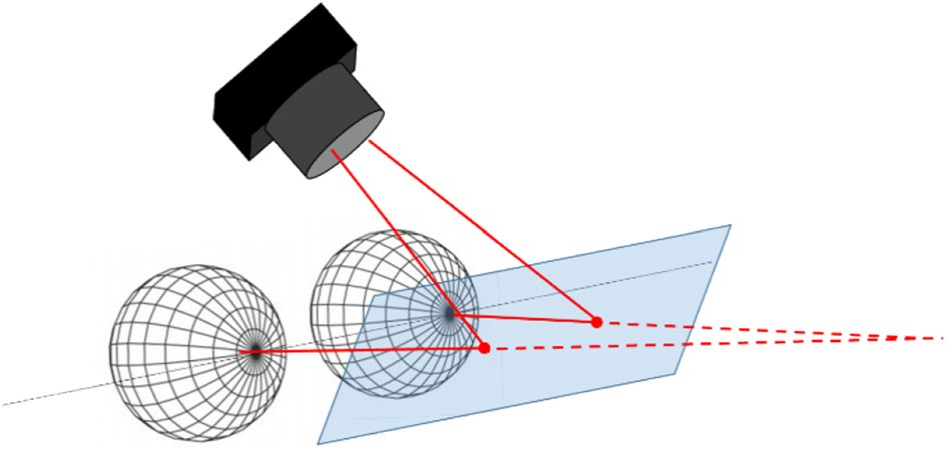
Gaze direction detection system. Each eye wears a scleral lens where a VCSEL is embedded. The VCSEL beams (solid line) are reflected by a beam splitter towards an IR camera placed above the eyes. The spot locations on the beam splitter are represented by disks and the direction of gaze by dashed lines.

Using a PSD enables, in a simple way, to detect several spots together (in contrast to complex image processing) so, that for each eye equipped with a laser pointer contact lens, it becomes possible to extract the vergence angle and then to deduce the sight direction knowing the eye’s position. Transparent PSD (transparent for the eye while sensitive (absorbent) for the laser beam; the wavelength being chosen in any case out of the visible range) could be manufactured, at the cost of a more complex optimisation of the substrate^27^. A simple alternative to a transparent PSD consists in using a beam splitter (BS) as shown in Fig. 1 together with an IR camera. This beam splitter is coated to reflect the IR beams generated by the two laser spots while being transparent for the eyes and coated to avoid unwanted reflections. Furthermore, it enables to materialize the beams (since it intercepts it, cf. Fig. 1 so that a single IR camera can be used to detect the spot motions on the BS surface. We use an IR camera (ELP Full HD 1920 × 1080 p) for our demonstration. In this case, a very elementary software can be developed either to detect the direction of sight or to track the eye trajectories.

In the following section, we present how to make the Cyclops lens and how the energy transfer towards the laser pointers is performed to obtain a very compact prototype.

## Material and methods

**Figure 2:**
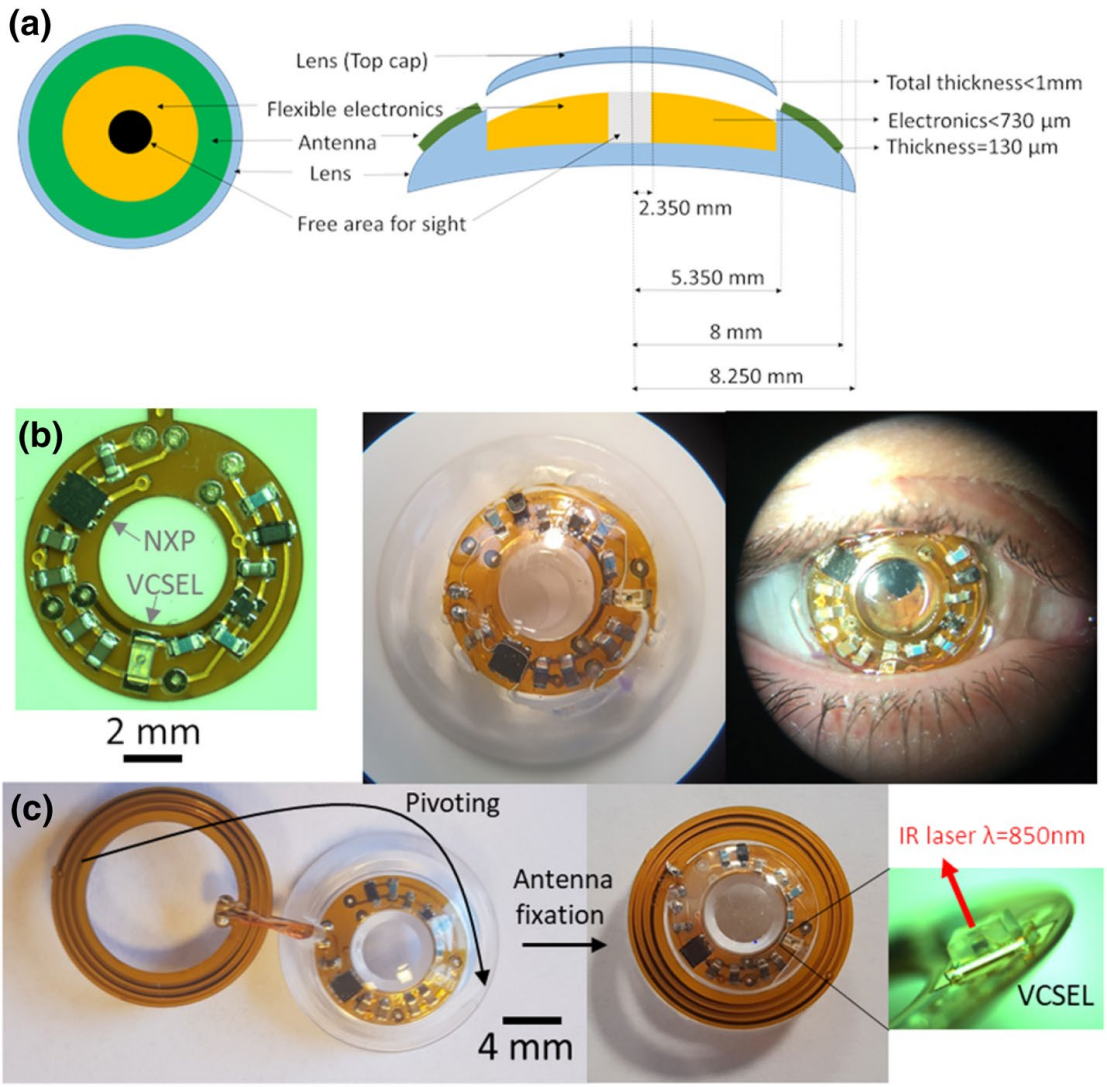
Successive steps from device to worn contact lens; (**a**) dimension and content of the contact lens, (**b**) sealed electronics: from left to the right, electronic circuit and components, encapsulated in the contact lens, Cyclops worn on a human eye. (**c**) top view of the cyclops lens with secondary antenna connected to electronics (insert : oblique view of the VCSEL).

The first part of our work of designing and fabricating the cyclops lens focus on the electronics used to trigger a vertical surface emitting laser (VSCEL) via a near-field power transfer-circuit. Figure 2 presents the electronic functions to be encapsulated into the Cyclops lens. It involves a harvesting and communication NFC (near field integrated circuit) followed by a tank capacitor to enable continuous energy to feed the rest of the circuit. For the Cyclops demonstrator, a flasher circuit is done with a free oscillator and a 850 nm VCSEL. To light periodically the VCSEL source, an oscillator enables a current pulse with a duty cycle of less than 10% of a period time of about a second to display a flashing detectable effect. It allows to decrease the time the VCSEL is on, to decrease the device energy consumption and the heating. The harvesting circuit is an NFC NTAG circuit NT3H2221 from NXP providing energy harvesting management for autonomous operation with regulated voltage output up to 10 mW. The communication function of the NFC chip has not been exploited here because not needed for eye tracking. However the addition of other sensors in contact lenses may need to communicate data. The human eye has been still considered as an attractive healthcare platform for continuous monitoring of biomarkers. The flasher uses an oscillator, based on a very low voltage Schmidt inverter (TI) as active device and a timing control with low duty cycling (less than 1%) and long period (about one second). The VCSEL has a threshold of 1.5 to 2 mA and an operating current of 3 to 4 mA potentially being able to deliver 0.5 mW at 850 nm. Due to dimensional constrains shown in Fig. 2a), we used thin electronics components and thin polyimide circuits. The flexible circuit, ordered from db electronics after design, is made up of a 25 µm polyimide layer, a 12 µm adhesive, 35 µm thick copper, coated with a protective resin (around 30 µm). Where the contacts appear, the copper is coated with 3–5 µm thick nickel and 50 nm thick gold. The flexible circuit is 130 µm thick due to the double copper layer. Before micro-components are transferred to the contact terminals, a silver conductive adhesive layer, Polytec EC 101, is applied to each of them. Once all the components are deposited, the adhesive is cured at a temperature of 120 °C for 1 h. The circuit is then ready for encapsulation.

The contact lenses used to encapsulate the VCSEL and associated electronics are scleral ones with a diameter of 16.50 mm, Fig. 2a. Such a type of contact lens no longer comes into contact with the cornea, but with the sclera (white part of the eye) thus providing a protective surface against potentially irritating elements for the eyes (dust, projections, irritating gas). This absence of contact with the cornea makes them extremely comfortable. They are steady (unlike usual contact lenses) when worn, which is a prerequisite regarding the application. Their large dimensions when compared to other contact lenses, and the possibility to use some of the space of a few hundred microns between the surface of the cornea and the inner part of the scleral lens, offer more volume for the electronics. For these reasons, the scleral lens is well suited for the integration of multiple electronic devices for different applications.

A customizable encapsulation for our device has been developed with Loctite 3301 glue to achieve a perfect sealing, the best optical properties in the pupil-free zone and to test the cyclops contact lens worn by human^20^. The “cavity” encapsulating the circuit was obtained by machining a PMMA puck. The prototype consists of two half-lenses. A half-lens receives the circuit and a half-lens closes the assembly via a gluing process performed by ISO 13485 certified scleral lens manufacturer. Figure 2b presents the contact lens encapsulated hermetically with its circuits and components. We observed a slight downward shift of the cyclops lens (Fig. 2a) with respect to the pupil centre when it was worn. This is due mainly to three factors; the conjunctiva morphology presenting a natural toricity (we used a spherical contact lens here), the weight (contact lens and embedded circuits) and the upper eyelid pressure (the thicker the lens, the more important it is). Despite this unexpected positioning, the lens was comfortable to wear and one could look through. In the future, the circuitry and contact lens design will be reviewed to address this issue. Various solutions are available, for instance, reducing the number of electronics components (limited to energy harvesting and laser functions), removing the packaging of the components, a better adaptation of the back surface to the corneal geometry of the user, by inducing a toricity on the inner surface of the contact lens, and reducing its thickness. Of course, a range of Cyclops contact lenses with different radii of curvature would have to be manufactured so that the lens can be fitted correctly to a particular eye physiology.

To provide a final contact lens with a satisfying design for a proof of concept, the flexible substrate with the components has been encapsulated on a flat surface, while the secondary antenna was reported on the front surface of the contact lens and fixed with tapes. The circuit and antenna have been connected by a wire going through a hole into the contact lens (see Fig. 2c). The secondary antenna has five turns with 0.3 mm width, with R = 4 Ω and L = 1.05 µH. Obviously, the antenna will be embedded into the contact lens in a next iteration, to prevent technical issues (electrical vias, comfort etc.), but this prototype allows us to demonstrate the proof of concept of a complete eye tracking system with a standard scleral contact lens (in terms of geometry). A key issue when encapsulating a light source is the energy consumption and autonomy of the encapsulated optoelectronic devices. In practice, this issue can be addressed in several ways, i.e. by incorporating a flexible battery in the contact lens as demonstrated in^28^ or by using harvesting techniques, the most common being the RF one^29,30^ which has been chosen here and detailed in the next section.

## Eye tracking prototype, description and operation

The cyclops contact lens is therefore made up of two main elements: the embedded optoelectronic circuitry and the secondary antenna. The full eye tracking system needs an external driving element, hosted by the eyewear with the primary antenna used to transfer energy (and possibly exchange data) and the optical tracking systems (camera).

**Figure 3:**
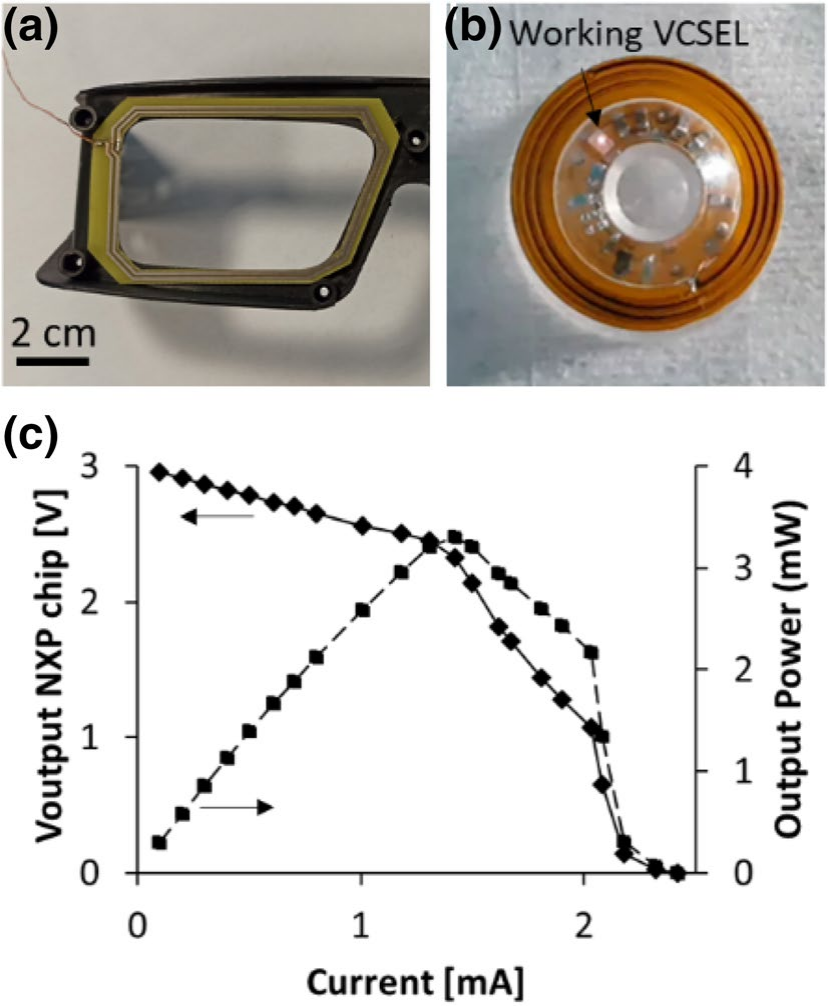
Transmitted RF Energy from the primary antenna to the NXP output (**a**) electronic primary antenna in the eyewear, a wire in the left corner connects it to a generator, (**b**) image of the directional flashing VCSEL of the smart lens. (**c**) Power harvesting capability of the NXP chip, with the output supply voltage (filled diamond) and the output power (filled square) as a function of the load current.

The primary antenna is designed to fit into the eyewear without impairing the vision. Its characteristics are L = 1.47 µH and R = 4 Ω for four turns on double face rigid circuit. It is coupled with the secondary antenna on the contact lens to transfer power by magnetic coupling. Several options are possible concerning the operating frequency. We have chosen 13.56 MHz (industrial, scientific and medical (ISM) band). This ensures a trade-off between the transmission range and efficiency, and the biocompatibility^30^. The primary antenna is itself connected by a wire to a generator (in future it will be connected to a battery packaged in the eyewear). In order to characterize the output power of the NXP chip, an input electrical power of 25 mW is injected into the primary spiral coil antenna. Primary and secondary spiral coil antennas are facing each other and 13 mm apart. Considering these two fabricated spiral coil antennas and the injected input power into the primary antenna, the voltage supplied by the NXP chip as a function of the load current has been measured, giving the output power capacity. Figure 3 presents the primary antenna embedded in the eyewear, the NXP chip output power and the emitting VCSEL (small bright dot).

The experimental results, Fig. 3c, are analysed to ensure that the NXP chip can power the VCSEL. The first part of the curve (from 0 to 1.4 mA) highlights the operation of the voltage regulator included in the NXP chip, the output voltage keeps stable in this operating range. The second part of the curve (up to 2.3 mA) shows a voltage drop as the load increases. The measurement shows that the power capacity of the NXP chip (considering the fabricated spiral coil antennas and the power injected into the primary antenna) is close to the 1.5 mA current threshold required by the VCSEL, but less than the nominal operating current of 3 mA. However, the low duty cycle (1% of 1 s period) of the timing control enables a continuous function. A 40 µF storage capacitor is charged by the NXP chip with a current of 1 to 2 mA (requiring less than one second), and powers the VCSEL with 3 mA for several ms during discharge, sufficient for laser emission. This also helps to limit the power injected into the primary antenna and the effect of RF waves in the human tissues. In parallel, we measured the minimum RF power required to make the eyetracker operating correctly (i.e. laser spot emitted is detected by the camera). We have found out that we needed only 5 mW of RF power injected into the primary antenna and 600 nW of output optical power of the VCSEL in order to have the system operating correctly, values which lead easily to compliance with the safety norms.

**Figure 4:**
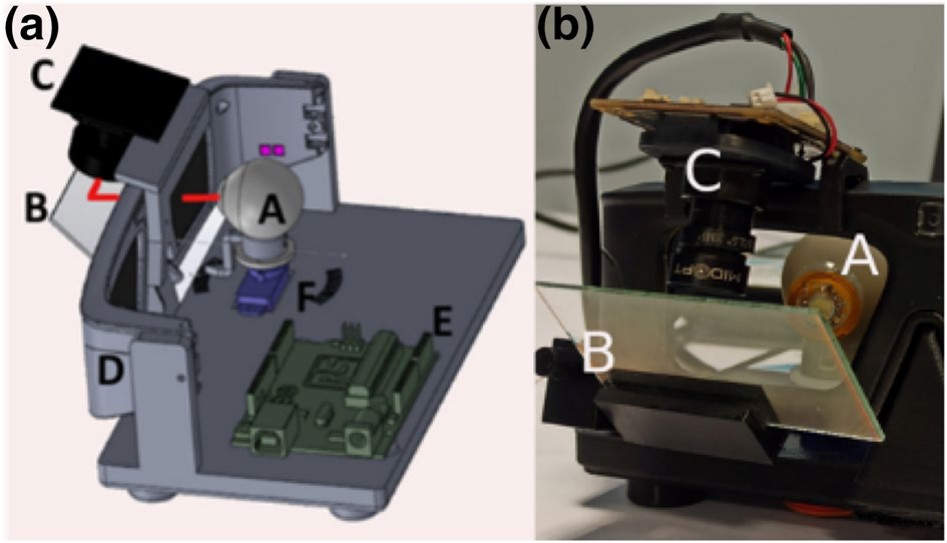
Eye tracking demonstrator (**a**) schematic of the overall system depicting the artificial eye (A) on which the cyclops lens is mounted, the servo-motor (F) and associated micro-controller (E) to control the eye direction, the eyewear (D) with the primary antenna, the beam splitter (B) reflecting the VCSEL beam (represented by a solid red line) towards the IR camera (C) (mounted on top of the eyewear). (**b**) A photo of the prototype.

As previously stated, the electronic eyewear includes an IR surveillance camera (ELP Full HD 1,920 × 1,080 p) and a beam splitter with custom coating on each surface as depicted Fig. 4. The camera is mounted on top of the eyewear. Mechanical holder allows adjusting the position and tilt of the beam splitter with the camera. The goal of the beam splitter is to reflect the VCSEL beam and to send it to the camera. The BS coating allows maximizing reflections at 850 nm and minimizing the ones in the visible range (enabling the Cyclops lens wearer to look comfortably through the system). As a result, a single spot corresponding to the beam position on the BS is imaged on the camera. The complete experimental platform is described in Fig. 4. The lens is placed on an artificial eye, 15 mm behind the eyewear (which corresponds to the usual eye-glasses distance). A servo-motor allows rotating the eye from − 20° to 20° around a vertical axis passing by the centre of the eye. This angular domain corresponds to the maximum field of view that would be practically explored by the eyes (i.e. in practice, if we need to observe something further than 20° we will turn the head rather than the eyes).

## Results and proof of concept

**Figure 5:**
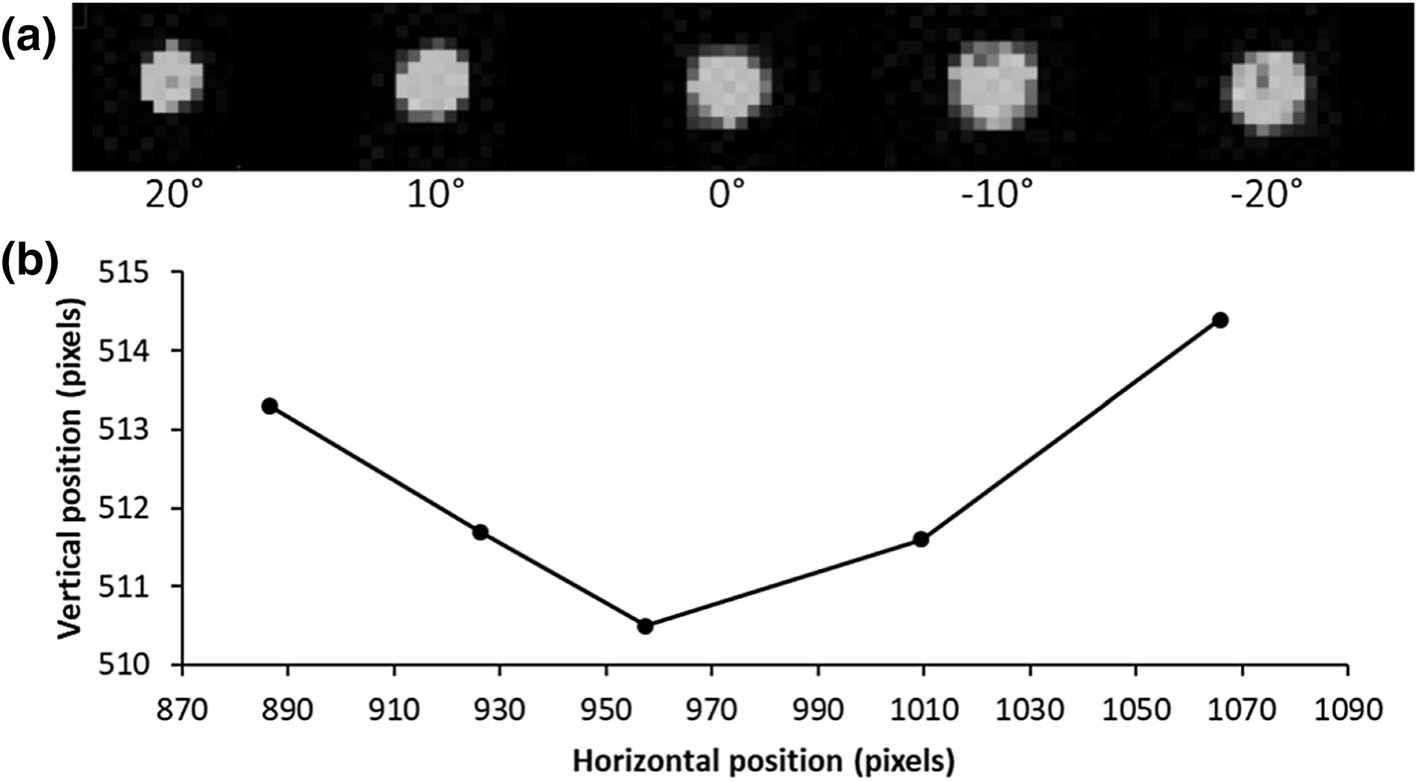
Results of the eye-tracker (**a**) Upper image representing successively the VCSEL spot recorded on the IR camera for 5 different orientations (− 20°, − 10°, 0°, 10°, 20°). Please note that it is a concatenated image of the spot in different positions (See Video S1). (**b**) The graph shows the VCSEL spot coordinates for the 5 previous positions.

Figure 5a represents the same image, five spots recorded by the IR camera for five different gaze directions (− 20°, − 10°, 0°, 10°, 20°). As it can be seen, the high directionality of the VCSEL beam allows obtaining a small round spot (approximately seven pixels of diameter) that is not distorted at the periphery. Thus, the coordinates of the spot’s centre (which represents the gaze direction) can be easily extracted with basic image processing techniques. In our device, the spots travels over 180 pixels between the two extreme positions (± 20°). This variation is roughly linear, Fig. 5b. A slight asymmetry can be observed which is due to the fact that the VCSEL is not positioned right in the middle of the eye and the beam splitter is slightly tilted with respect to the spectacles frame. However, this deviation can be easily corrected with proper calibration. Therefore, if we assume an error of one pixel when calculating the coordinates of the spot centre, we can say that our device has a 0.2° accuracy (or 0.3° if we were to consider both x/y directions). which is almost one order of magnitude better than high end commercial wearable eye trackers (although it come close to the 0.6° accuracy reported by Tobii Pro glasses for small eccentricities (± 15°)).Obviously, a variety of environmental factors (such as lightings and slippage of the beam detector) could reduce the eye tracking performances in real conditions However, this preliminary result is all the more interesting that it was achieved with off-the shelves components without trying to optimize the eye tracker accuracy).The purpose of our study was a proof of concept of the interest of using a contact lens laser pointer as an eye tracker. A careful optimization of the device (e.g. CCD resolution) or, as written previously the use of PSD, could allow achieving yet a higher resolution.

**Figure 6:**
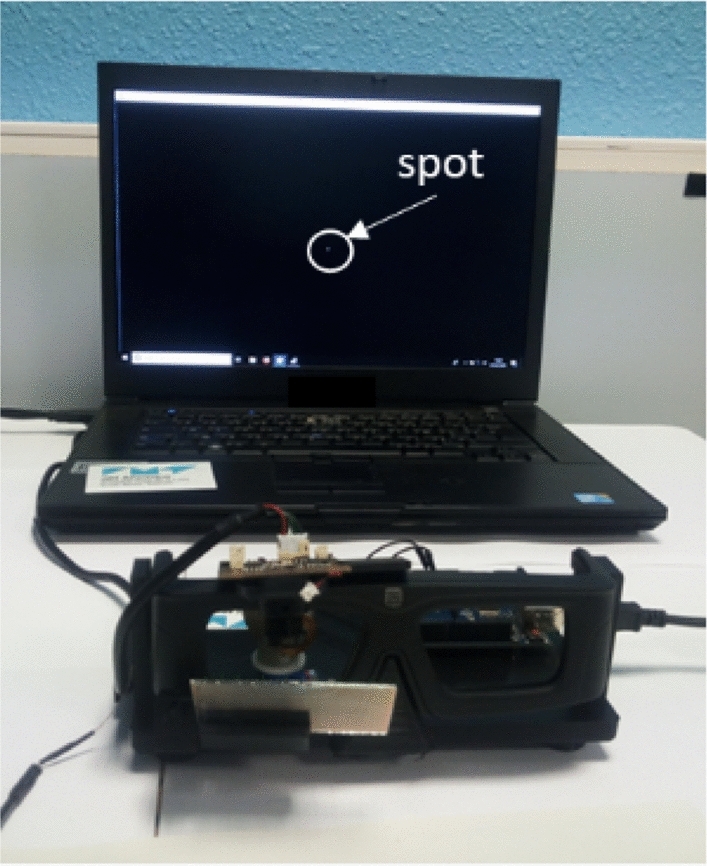
The Cyclops prototype in the foreground, with the camera mounted on the eyewear and connected to a PC in the background (we can notice the white spot). See Video S1.

Figure 6 gives an overview of the optical demonstrator, while the related attached video shows how the system works dynamically in real time. In the first part, we can observe the correlation between the eye robot rotation (azymuthal) and the spot motion on the PC screen in the background of the scene, such as detected by the camera. In the second part, we can observe the cyclops contact lens motion through the BS in a front view.

The use of a laser close to the eye inevitably raises some health concerns. In perfect conditions of use, one part of the eye that the VCSEL beam may interact with is the eyelid during blinking.

If we consider that most people blink around 15 times a minute (150 ms per blink in average), this means that during a 1 h use, the eyelid will be exposed to the VCSEL beam approximately 900 times. If we consider that the VCSEL beam will always hit exactly the same spot, a laser at 850 nm could potentially trigger some photosensitive reaction or even burn depending on the power emitted and its divergence. In our set-up, the VCSEL was first of all chosen for its dimensions, wavelength and availability and could potentially present a risk if used at full power. However, in the case were the VCSEL was used at minimum power, the associated radiant exposure (0.5 kJ/m^2^) was, according to regulations (BS EN 60825-1:1994), significantly below the maximum permissible exposure for the worst case scenario (4 kJ/m^2^ considering that the subject keeps his closed all the time). Another potential risk associated to the laser beam is the back scattering, i.e. if the laser is reflected towards the retina. The current device is not optimized to avoid such reflections but fortunately, this can be easily avoided with classical optical engineering techniques (e.g. coatings).

## Conclusion

We have demonstrated the principle and feasibility of a new generation of eye trackers, using a laser pointer embedded into a wireless scleral contact lens. This solution which materializes the gaze direction simplifies the detection in terms of computing calculation, and improve accuracy thanks to the low divergence and high beam quality of VCSELs compared for instance with light emitting diodes. In particular, we have shown that a low RF energy and the associated small emitting optical power is enough to be detected by a standard camera, located a few cm far from the eye.

In ideal conditions (no lens/eyewear slippage), this accuracy depends mainly on the spot size and the photo-sensor resolution. For a proof of concept, we used a camera with an IR depends mainly on the spot size and the photo-sensor resolution. PSD is an alternative, its advantage is that there is no need for imaging optics and therefore the detection system can be very thin. In contrast, the detection function is limited to a power peak detection. It was not—our objective to compare both options. It has been done, for instance, for a similar configuration to measure laser angular deviation in refractometers^31^ and have been shown to enable a very accurate spot detection. As previously stated in the results, section, another parameter that can affect the accuracy is the contact lens motion. We wish to emphasize here the advantage of using a scleral contact lens to implement cyborg contact lenses in the future. Since the late 1800s considerable progress have been made in scleral lens design and manufacturing. Thick scleral lenses exist (e.g. lenses with high refractive power or electronics one), and techniques to stabilize them have been demonstrated. Modern scleral lenses offer unprecedented stability, e.g. 100 microns shift over an hour (see^32^). Such a shift would lead to a maximum error of 4 arcmin on the calculation of the angle made by the VCSEL beam.

The fact that the lens appears mispositionned in Fig. 2, is due mainly to two factors: weight and the fact that the lens had not been specifically adapted for the eye. Regarding the first point, we only used here off the shelfs component to make a lens whose geometry is in agreement with scleral lens standards. Weight could be easily reduced with custom components. Regarding lens fitting, a range of cyclops lens with different radii of curvature would have to be manufactured so that the lens can be fitted correctly to a particular eye. Therefore, when correctly fitted, a lens shift should cause a maximum error of few arcmin. A much more serious problem than lens slippage is a shift of the beam detector (just like the shift of eye tracking glasses can be an issue).

Concerning the acceptability and safety, to ensure RF safety in practical uses, the impact of RF exposure in the human tissues needs to be investigated, and particularly to verify if the specific absorption rate (SAR) satisfies specific limits^31,33^. ICNIRP^30^ and IEEE^33^ might have slight differences in setting the regulations for RF safety levels. The SAR performance of the cyclops eye-tracking system has not been evaluated yet, however, it will not lead to safety issues comparing it to similar ones presented in literature^29^. In the work^29^, the same power transfer mechanism, the same frequency for wireless power transfer (13.56 MHz) and very similar distances between a primary antenna on eyeglasses and a secondary antenna on the contact lens were used. The power received at the contact lens was higher than in our work and the SAR simulated value was almost 100 times smaller than the SAR limit of 2 W/kg proposed by ICNIRP^30^. Similarly, before humans could wear a fully functional system, several tests (oxygen permeability, cytotoxicity, irritation, tolerance) needs to be carried out to obtain a clinical certification.

Concerning the key future developments, they concern mainly the flexible electronics and stretchable transparent antenna compatible with a moulding process. Park et al.^17^ demonstrated how electronics and antenna conforming the curved shape of soft contact lenses operated reliably during mechanical deformations, including bending and stretching, making them compatible with large-scale mass production. The second challenge for cyborg lenses, expected to include more and more computing power, is the heating dissipation and presence of hotspots (e.g. antenna, ASIC, laser). In vivo tests monitoring the temperature change on rabbit’s eyes provided substantial promises of future smart contact lenses for non-invasive health care using human eyes^17^. However, at this early stage, no specific regulations exist. A physiological acceptance between 35° (open eye) and 37° (close eye) is cautiously considered. This point will be investigated further in the future. In that frame, using a scleral contact lens benefiting from a tear tank between the cornea and the embedded circuit is a real advantage.

## Supplementary information

Supplementary Video
